# Critical Review of the Cross-Links Between Dietary Components, the Gut Microbiome, and Depression

**DOI:** 10.3390/ijms26020614

**Published:** 2025-01-13

**Authors:** Nidesha Randeni, Baojun Xu

**Affiliations:** Food Science and Technology Program, Department of Life Sciences, BNU-HKBU United International College, Zhuhai 519087, China; nidesha.randeni96@gmail.com

**Keywords:** diet, gut health, dietary patterns, mental health, neurotransmitters

## Abstract

The complex relationship between diet, the gut microbiota, and mental health, particularly depression, has become a focal point of contemporary research. This critical review examines how specific dietary components, such as fiber, proteins, fats, vitamins, minerals, and bioactive compounds, shape the gut microbiome and influence microbial metabolism in order to regulate depressive outcomes. These dietary-induced changes in the gut microbiota can modulate the production of microbial metabolites, which play vital roles in gut–brain communication. The gut–brain axis facilitates this communication through neural, immune, and endocrine pathways. Alterations in microbial metabolites can influence central nervous system (CNS) functions by impacting neuroplasticity, inflammatory responses, and neurotransmitter levels—all of which are linked to the onset and course of depression. This review highlights recent findings linking dietary components with beneficial changes in gut microbiota composition and reduced depressive symptoms. We also explore the challenges of individual variability in responses to dietary interventions and the long-term sustainability of these strategies. The review underscores the necessity for further longitudinal and mechanistic studies to elucidate the precise mechanisms through which diet and gut microbiota interactions can be leveraged to mitigate depression, paving the way for personalized nutritional therapies.

## 1. Introduction

The intricate relationship between diet, the gut microbiome, and mental health has emerged as a focal point in contemporary biomedical research. This triad of components is the basis of the gut–brain axis—the bidirectional communication network between the enteric nervous system (ENS) and the CNS that “talks” back and forth and integrates information from the gut and the brain to regulate bodily functions and mental health. The gut–brain axis encompasses several different pathways (immunological, hormonal, and neurological), all contributing to GI functions and the mental state of individuals [1].

Dietary influences play a major role in shaping the gut microbiome—the ecological community of billions of microorganisms residing within the gastrointestinal (GI) tract [2]. These microbes are not only present passively but also play active roles in several physiological processes, like the modulation of immune response, production of neurotransmitters, and even synthesis of some essential metabolites. There are several kinds of dietary intervention that cause changes in the diversity and composition of the gut microbiota [3], with some foods being more beneficial for enhancing certain bacterial groups while others cause an imbalance called dysbiosis, which is known to be associated with several diseases [4].

Research on depression, which is a widespread and debilitating mental disorder, has revealed that changes in the gut–brain axis may be implicated in the condition. There is a growing body of evidence indicating that gut microbiota may influence functions of the brain and behaviors through the synthesis of neuroactive substances, changes in the immune response, and the modification of the integrity of the gut barrier. All these factors are important and contribute to the understanding of the disease mechanisms underlying depression, as well as providing new approaches for treatment by manipulating the gut microbiome [5].

The objective of this review is to conduct an integral and objective survey of all literature available on the links connecting depression, gut microbiota, and diet. In particular, we will elucidate the mechanisms within the gut–brain axis—particularly focusing on neurotransmitter production, immune response, and microbial byproduct composition—that we hypothesize can explain how dietary interventions can modify gut microbiota to improve mental health. This review will be framed within the context of recent advances in the field, highlighting key studies and identifying gaps in the knowledge that warrant further investigation.

## 2. Gut Microbiome and Depression

### 2.1. Composition and Function of the Gut Microbiome

The gut microbiome comprises numerous microorganisms that play critical roles within the host. The gut microbiota is dominated by bacteria, with the majority belonging to four dominant phyla: *Firmicutes*, *Bacteroidetes*, *Actinobacteria*, and *Proteobacteria* [6]. These phyla are interconnected to form a microbial community, which is considered unique for each species in the community because of the effects of the host’s genetics, diet, age, geography, and lifestyle [7]. *Firmicutes* is the major phylum of the gut microbiome and contain several families, such as *Lactobacillaceae*, *Clostridiaceae*, and *Ruminococcaceae*. Microorganisms belonging to *Firmicutes* are critical for the fermentation of complex plant materials into SCFAs such as butyrate, which has been shown to possess anti-inflammatory properties while also supporting the health of intestinal tissues [8]. In particular, *Bacteroidetes*, including species from the genus *Bacteroides*, is involved in the breakdown of protein and polysaccharide complexes in the gut into smaller units for utilization by the host and other microbes. These organisms are important for the fermentation of carbohydrates and the production of SCFAs important for metabolic health, such as acetate and propionate [9]. While members of the *Actinobacteria* phylum, such as *Bifidobacterium*, are quite common in the gut microbiome of young children, this group is also significant in adult microbiomes. *Bifidobacterium* species include patented probiotic products that have effects on the immune system and prevent the colonization of the gut by pathogenic organisms [10]. Although not as abundant as the other major phyla, *Proteobacteria* comprises *Escherichia*, *Salmonella*, and *Helicobacter* species that play a role in the gut. Whereas some *Proteobacteria* are non-pathogenic members of the skin microbiota, others are opportunistic pathogens that cause dysbiosis at elevated population densities. *Proteobacteria* species promote inflammation and can be upregulated in diseases such as IBD [11].

In addition to bacteria, the gut microbiome also includes archaea, viruses, fungi, and protozoa [12]. One of the archaea species is *Methanobrevibacter smithii*, involved in methane production, and some others are involved in specific digestive processes. Many of the components of gut virome are bacteriophages, viruses that infect bacteria and that function as population control agents. Although they are fewer in number, fungi are also involved in maintaining gut health, but when they start to proliferate, problems occur, as seen in Candida dysbiosis [13].

Every organism in an individual microbiome is connected with the other organisms to form associations that are considered significant for the host organism’s health status. The proportion of these microbial communities is essential because the disruption of the balance of the microbiome, known as dysbiosis, is associated with many diseases, for instance, metabolic, autoimmune, and psychiatric disorders.

### 2.2. Gut Microbiome Dysbiosis and Depression

**Figure 1:**
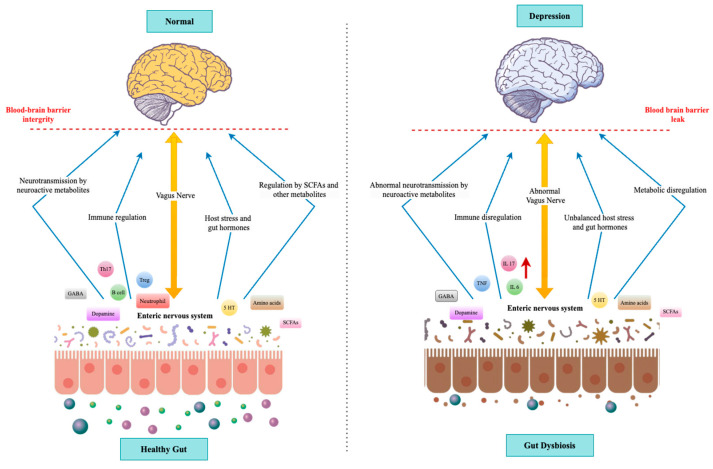
Gut dysbiosis in depression.

The connection between depression and gut microbiome dysbiosis is a topic that has garnered progressively increasing scientific attention concerning its usefulness in explaining how imbalances in the microbial populations in the human gut can cause mental health disorders. Dysbiosis is defined as a reduction in beneficial bacteria (for example, *Bifidobacterium* and *Lactobacillus*) and an increase in potentially pathological bacteria (some strains of *Proteobacteria* and *Clostridia*) [14]. This imbalance can disrupt the integrity of the intestinal barrier, promote immune activation, and create a state of low-grade inflammation, which is believed to affect depressive-like behavior via bidirectional communication between the gut and the brain (Figure 1).

This is supported by one of the effects of dysbiosis, in which dysbiosis triggers the hypothalamic–pituitary–adrenal (HPA) axis, which is very crucial in the stress response pathway. An imbalance in microbiota results in enhanced permeability of the intestinal barrier, or “leaky gut”, and causes the liberation of bacterial endotoxins such as lipopolysaccharide (LPS). These endotoxins act as immunostimulants, thus increasing the production of pro-inflammatory cytokines. These cytokines appear to modulate neurotransmitter function and neural plasticity, both factors related to mood regulation [15]. The dysbiotic gut also impacts the levels of neuroactive metabolites that function in the brain. A healthy gut microbiota synthesizes hormones, including serotonin precursors and SCFAs such as butyrate, necessary for modulating brain health. An ideal microbiome makes up to 90% of the body’s serotonin, which is responsible for mood. These metabolites include SCFAs, the production of which decreases in dysbiosis—a state that interferes with these neurotransmitter pathways and influences the development and severity of depression [16]. In addition, dysbiosis is linked with the overproduction of reactive oxygen species (ROS), which influence inflammatory processes in the gut and within the brain. Furthermore, an imbalance in the gut microbiota may affect neurogenesis, causing a decline in the generation of neurons in the hippocampal part of the brain [17]. In particular, the data also imply that dysbiosis affects the endocannabinoid system, regulating such processes as emotions, stress, and anxiety. Some of the gut microbiota directly modulate endocannabinoid tone, affecting the brain and mood. This homeostasis is imbalanced in dysbiosis, with possible dysregulation of mood and increased levels of depressive symptoms [18]. With depressive-like behavior, experiments on in vivo serotonin, dopamine, norepinephrine, 5-HIAA, 3-MT, HVA, and MHPG metabolic reduction have revealed a gut microbiota shift [19]. Overall, gut microbiota imbalance plays a role in the disruption of the anti-inflammatory and anti-neurotoxic milieu; it participates in mood regulation through numerous processes. Knowing this connection means there are new options for the use of dietary and probiotic interventions targeting the restoration of normal gut microbiota, possibly for the prevention and treatment of depression.

Diet is the main factor associated with dysbiosis. It is worth noting that the human gut microbiome is very dynamic and sensitive to the types of foods eaten, where various diets and types of foods support the growth of particular microbial communities. A diet rich in fiber helps in the growth of friendly bacteria that produce SCFAs, which are important in gut health and immune defense [20]. On the other hand, the Western diet, associated with the high consumption of saturated fats and sugars, can have negative health effects that translate to an increase in inflammation-promoting bacteria and a decrease in human bacterial counts, otherwise known as dysbiosis [21]. This imbalance can disrupt the gut barrier function, allowing for the translocation of microbial products into the bloodstream and triggering systemic inflammation. Further, low nutrient density, as found in foods with few vitamins and minerals, affects microbial metabolism and decreases the synthesis of important metabolites that are beneficial for host physiology [2]. In contrast, the oral intake of red ginseng lowered the levels of depression in mice and stabilized changes in the gut microbiota during anxiety and depression [22]. In this regard, the type and contents of food consumed decide the nature of the gut microbiota, and a substandard diet tends to create dysbiosis and result in various health complications, including metabolic disease, inflammation, and other mental health disorders such as depression.

### 2.3. The Gut–Brain Axis

The gut–brain axis is a two-way communication system connecting the CNS with the ENS through neural, hormonal, and immune system messengers. This bidirectional system is capable of coordinating messaging between the brain and GI system and, therefore, has a significant effect on the physical and psychological well-being of the body [23]. Specifically, it has been found that the gut microbiota and its bidirectional communication with the brain affect mood [24], mental health, decision-making [24], and even preference for sweetness [25]. Factors such as the ability of the gut microbiota to use the host and influence it to act in a manner favorable for the microbiota via reliance or local manipulation seem probable. In this case, the ways the host perceives and activates their brain and body may be modulated by gut flora “by force”.

**Figure 2:**
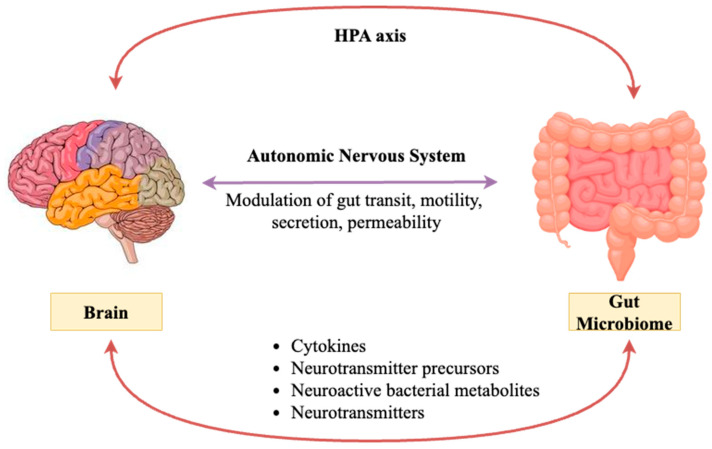
The gut–brain axis.

The vagus nerve is believed to be the primary neural pathway in the gut–brain axis, mediating communications between the brain and the gut. It plays an important role in digestion, mood, and controlling inflammation [26]. The gut sensory neurons communicate through enteric nerves about the state of the gut to the brain, while motor neurons carry signals from the brain to the gut for the purpose of motility and secretion processes [5]. Furthermore, the ENS, also known as the “second brain”, consists of a massive network of neurons operating largely independently from the CNS, though modulated by it via the vagus nerve and other autonomic pathways [27] (Figure 2). Other important components of the gut–brain axis are hormones and neuropeptides. These gut hormones—ghrelin, leptin, and peptide YY, among others—influence appetite, metabolism, and even mood [28]. For example, the “hunger hormone” ghrelin, besides stimulating appetite, influences mood and cognitive functions, acting via the hypothalamus and other encephalic regions [29]. Furthermore, the involvement of the HPA axis, specifically stress hormones such as cortisol, interferes with gut function and microbiome composition, thus having impacts on mental health [30] (Figure 2). The gut and the brain interrelate through the immune system, wherein GALT plays a major role [31]. Moreover, inflammatory cytokines produced within the gut may cross the blood–brain barrier or signal through the vagus nerve to affect brain functions, influencing the development of mood disorders such as depression [32]. Dysbiosis leads to increased intestinal permeability known as “leaky gut”, which allows bacterial endotoxins to enter the bloodstream and induce systemic inflammation. This inflammation can then impact brain function and behavior [33].

The gut microbiota produces metabolites such as SCFAs and neurotransmitters (e.g., serotonin, GABA) that can modulate brain function. Serotonin, for instance, is predominantly produced in the gut, and its levels are influenced by the gut microbiome composition. Microbial metabolites can affect the integrity of the blood–brain barrier and modulate neuroinflammation, influencing mental health outcomes [34]. Serotonin, or 5-hydroxytryptamine (5-HT), is a key neurotransmitter involved in regulating mood, cognition, and GI function. Approximately 90% of the body’s serotonin is synthesized in the gut, predominantly by enterochromaffin cells, with a smaller proportion produced by neurons in the ENS [35]. Certain bacteria, such as *Enterococcus*, *Streptococcus*, and *Escherichia* species, can produce metabolites that influence enterochromaffin cells to increase serotonin production. GABA is the primary inhibitory neurotransmitter in the CNS, reducing neuronal excitability and playing a role in stress responses and mood regulation [36]. Certain gut bacteria, including *Lactobacillus* and *Bifidobacterium* species, can produce GABA. These bacteria convert glutamate, an excitatory neurotransmitter, into GABA through the action of glutamate decarboxylase [34]. GABAergic signaling in the brain is essential for regulating anxiety, stress, and mood. The dysregulation of serotonin levels in the gut and alterations in gut microbiota composition that affect GABA production may contribute to psychiatric conditions such as depression and anxiety disorders [34].

The gut–brain axis is significantly influenced by the immune system through mechanisms involving cytokine modulation, intestinal barrier integrity, and microglial activation. The gut microbiota interacts with the immune system, affecting both gut and brain health primarily by influencing cytokine production [37]. Cytokines are small proteins released by immune cells that play a key role in cell signaling, particularly in immune response. Dysbiosis can lead to the overproduction of pro-inflammatory cytokines, which can cross the blood–brain barrier or signal through the vagus nerve, promoting neuroinflammation and contributing to mood disorders such as depression [38]. Conversely, beneficial bacteria like *Bifidobacterium* and *Lactobacillus* promote the production of anti-inflammatory cytokines such as IL-10, supporting immune homeostasis [39]. Gut bacteria can also influence the expression and function of tight junction proteins that regulate intestinal permeability. Dysbiosis also compromises intestinal barrier integrity, resulting in a “leaky gut”, allowing endotoxins like LPS to enter the bloodstream and trigger systemic inflammation. This inflammation can activate microglia, the brain’s immune cells, leading to neuroinflammation, a hallmark of neuropsychiatric disorders. These interactions underscore the potential of targeting the gut microbiota for therapeutic interventions in improving gut and mental health [40].

### 2.4. Metabolites Produced by Microorganisms and Their Physiological Roles

The term microbial-derived metabolites refers to the biochemical compounds that are formed by the gut microbiome as it ferments fibers, proteins, and other substances in food. These compounds, which include SCFAs, bile acid derivatives, indoles, and neurotransmitter precursors, among others, have many physiological functions and are utilized outside of the gastrointestinal tract; they affect immunity, metabolism, and inflammation, as well as mental health. They act as crucial mediators integrating the gut with all other systems, including the brain, via the gut–brain axis. In addition, it has been established that the gastrointestinal tract has numerous endogenous gut hormones such as leptin, ghrelin, cholecystokinin, and glucagon-like peptide-1 (GLP-1). The mentioned hormones also include neuropeptide Y, connected to mood, anxiety, and the immunological system, as well as peptide YY, originating from two pancreatic polypeptides that promote satiety and enhance glucose balance and behavior [41].

SCFAs that are produced as a result of the bacterial fermentation of dietary fiber include acetate, propionate, and butyrate. These metabolites are important in the gut, as they enhance barrier function by promoting tight junctions and the secretion of mucus. SCFAs exert an anti-inflammatory effect and immunomodulatory function by inducing T regulatory cells (Tregs), which curb excessive immune responses and thereby help in controlling inflammation throughout the body [42]. Moreover, SCFAs upregulate the levels of the enzyme tyrosine hydroxylase, which regulates the rate at which the biosynthesis of catecholamines, including dopamine, adrenaline, and noradrenaline, occurs and also upregulates tryptophan 5-hydroxylase 1, which is a critical enzyme involved in producing the neurotransmitter serotonin [43]. Tryptophan, an indispensable dietary amino acid, is a source of numerous important metabolites, including serotonin, that are crucial for neuroendocrine signaling. The use of tryptophan to produce various metabolites, including tryptamine, kynurenine, and indoles, in particular, is attributable to a specific gut microbiome [44]. Tryptophan metabolites reach the CNS through the circulation or the vagal afferent pathway and, therefore, play an important role in neuroendocrine and neuroimmune activity [45].

The primary bile acids synthesized in the liver are converted by gut microorganisms into secondary bile acids that play a key role in lipid catabolism and the maintenance of cholesterol levels. These bile acids altered by microorganisms are also involved in cellular signaling and bind to receptors such as farnesoid X receptor (FXR) and G-protein coupled bile acid receptor 1 (TGR5), thereby modulating glucose metabolism, energy balance, and immune responses. In addition, bile acid metabolites help regulate inflammation, as certain bile acid derivatives can inhibit pro-inflammatory pathways, reducing the risk of chronic inflammatory diseases [46].

Kynurenine is another tryptophan metabolite, and *Lactobacillus* taxa regulate its production [47]. In order to decrease host kynurenine metabolism, *Lactobacilli* create hydrogen peroxide, an ROS that inhibits the production of the enzyme indoleamine-2,3-dioxygenase (IDO1). In the GI tract, IDO1 contributes to the synthesis of kynurenine from tryptophan [48]. The reduction in *Lactobacillus* brought on by stress lessened the inhibition of IDO1 mediated by hydrogen peroxide in a rat model of chronic varied stress, which increased the synthesis of kynurenine from tryptophan [49]. It has been demonstrated that kynurenine, which crosses the blood–brain barrier, causes neuroinflammation and neurodegeneration, which are also linked to depression and Alzheimer’s disease [47].

Derived from the bacterial metabolism of the amino acid tryptophan, indoles play a critical role in modulating gut barrier integrity, reducing gut permeability, and influencing immune responses. While the generation of kynurenine and serotonin from tryptophan is mostly dependent on gut microorganisms, indole synthesis is entirely microbe dependent because only specific microbes produce the tryptophanase enzyme needed to produce these chemicals from tryptophan [50]. Indole derivatives like indole-3-propionic acid (IPA) act as antioxidants and have neuroprotective effects. These metabolites also engage with the immune cell aryl hydrocarbon receptor (AhR), aiding in achieving immune homeostasis and reducing tissue inflammation. Indoles’ role in immune modulation, gut health, and barrier function underscores their significance in ensuring there is neither “leaky gut” nor systemic inflammation, which contribute to mood dysregulation and other disorders [51].

**Figure 3:**
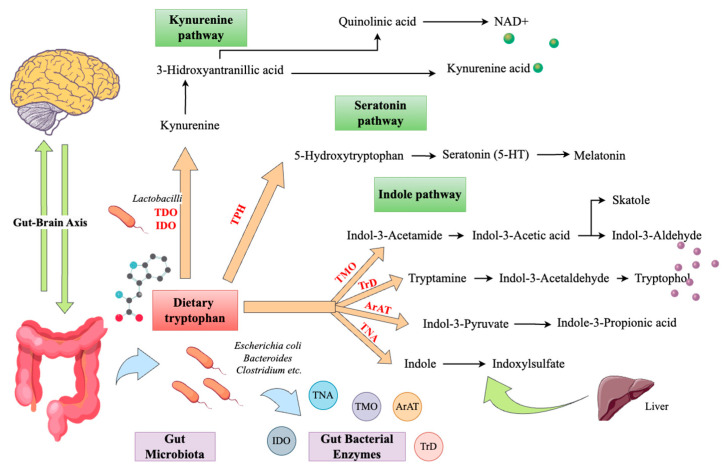
Neuroactive metabolites produced by the gut microbiome.

As shown in Figure 3, tryptophan is essential for the synthesis of serotonin, kynurenine, and indoles. Ingesting foodstuffs containing tryptophan, such as turkey, eggs, and dairy products, may serve to enhance serotonin levels. In addition, it is thought that dietary carbohydrates may further increase the level of tryptophan absorption by elevating insulin levels, which may suppress other amino acid competition [52]. This amino acid can be found in meat and dairy products, nuts, and soy products [53]. The proper levels of tyrosine are critical for the production and regulation of dopamine levels. It has also been argued that the consumption of many antioxidant-rich foods, including fruits and vegetables, can help shield neurons that generate dopamine from oxidative damage and stress [54]. GABA is produced when sufficient amounts of dietary glutamine are present. Foods such as nuts, seeds, and a few other fermented foods also help in GABA synthesis [55].

## 3. The Impact of Dietary Components on the Gut Microbiota

### 3.1. Carbohydrates

**Figure 4:**
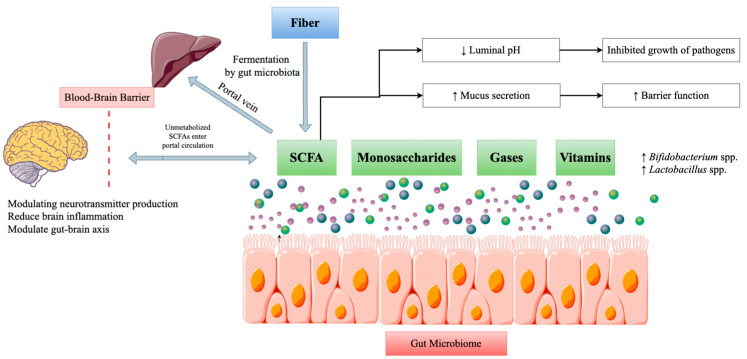
The function of dietary fiber in connection with brain function and gut microbiota. ↑, increase; ↓, decrease.

Carbohydrates are a major component of the human diet and significantly alter the activity and composition of the gut microbiota. The types of carbohydrates consumed, their fermentation by gut bacteria, and their impact on microbiome diversity are crucial factors in maintaining gut and overall health. Considering their chemical composition and digestibility, carbohydrates form two categories: simple and complex [56]. Unlike simple carbohydrates, many complex carbohydrates and fibers reach the colon, where gut bacteria ferment them, since they cannot be broken down in the small intestine. The breakdown of fibers depends on the breakdown of carbohydrates by the intestinal microbiota. Fibers are composed of resistant starches; non-digestible oligosaccharides, including raffinose, stachyose, oligofructose, and inulin; and non-starch polysaccharides such cellulose, hemicellulose, glucans, gums, and pectins [57]. This fermentation process has significant implications for gut microbiome diversity and function [58]. Gut bacteria produce monosaccharides, specific gases (such as carbon dioxide and methane), and short-chain fatty acids (SCFAs) like butyrate, propionate, and acetate through the saccharolytic fermentation of food fibers and resistant starches. SCFAs, with their anti-inflammatory properties, provide colonocytes with the energy needed, enhance gut barrier function, and influence host metabolism [59]. As illustrated in Figure 4, SCFAs like acetate and propionate are absorbed by the blood and move to the liver through the portal vein. While a significant portion of SCFAs is utilized by colonocytes and the liver, some unmetabolized SCFAs may enter the systemic circulation and exert effects on other tissues and organs like the brain, influencing brain function, neurotransmitter production, and brain inflammation [60].

The types and amounts of carbohydrates consumed can significantly influence the gut microbiome’s diversity and composition [61]. Diets that have high fiber contents encourage the development of good bacteria, like *Bifidobacterium* and *Lactobacillus* species, which have been acknowledged for their ability to enhance health. These bacteria utilize dietary fiber to produce SCFAs, contributing to a healthy gut environment and supporting immune function [62]. By encouraging the growth of harmful bacteria and decreasing the number of helpful microorganisms, diets heavy in refined carbohydrates—such as sugars and processed grains—can have a detrimental effect on microbiome diversity. This shift can lead to dysbiosis, associated with various health issues, including obesity, diabetes, and inflammatory bowel disease (IBD) [63]. Animal models have shown that the Western diet, which contains relatively little fiber, reduces the amount of *Bifidobacterium* and the diversity of the gut microbiota. [64]. It has been demonstrated that gut dysbiosis can be improved in rats fed a diet enriched with high-fat and sucrose by decreasing the *Firmicutes*-to-*Bacteroidetes* ratio and increasing the amount of *Lactobacillus* sp. [65].

### 3.2. Protein

**Figure 5:**
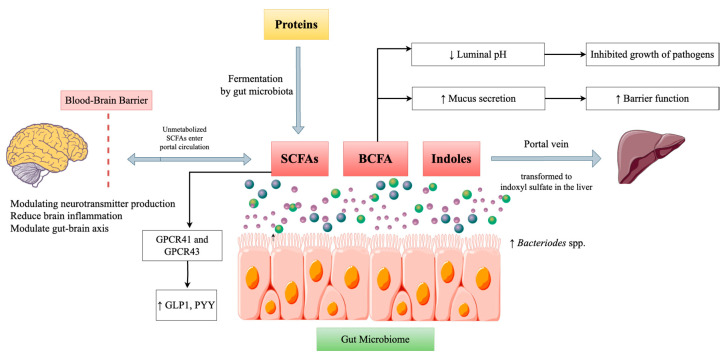
The role of proteins in the gut microbiome and brain function. ↑, increase; ↓, decrease.

Proteins are essential macronutrients that are necessary for several physiological functions, including immunological response, growth, and repair. The digestion and fermentation of dietary proteins by gut microbiota produce several byproducts, and they have the capacity to greatly impact microbial composition and gut health. Peptides and amino acids are absorbed into the bloodstream through the intestinal epithelium in the small intestine, where most protein digestion occurs. However, a fraction of dietary proteins and peptides escape digestion and reach the colon, where they undergo microbial fermentation [66]. In the colon, undigested proteins and peptides are broken down by gut bacteria, producing various byproducts that impact gut health and microbial composition [67]. Gut bacteria metabolize amino acids to generate a variety of compounds, such as SCFAs, branched-chain fatty acids (BCFAs), and gases like hydrogen and methane (Figure 5). Key amino acids involved in these processes include indoles, glutamine, lysine, and arginine [68]. Ammonia, amines (e.g., putrescine, cadaverine), phenolic compounds (e.g., p-cresol), and hydrogen sulfide can also be produced as a result of protein fermentation. These metabolites can have toxic effects on the gut epithelium and are associated with a higher risk of diseases such as colorectal cancer and IBD [66].

The type and quantity of dietary protein ingested play an important role in altering the diversity and composition of gut microbiota. Diets that consist of more animal proteins tend to promote the growth of protein-fermenting bacteria such as *Bacteroides* and *Clostridia* species, among others, and suppress the growth of carbohydrate-fermenting bacteria such as *Bifidobacterium* and *Lactobacillus*. This particular shift may lead to the generation of more harmful by-products and fewer beneficial SCFAs [69]. Diets rich in plant proteins, especially those from pulses and cereals, have been linked to a healthier and more stable gut microbiome. In addition, plant proteins often contain dietary fiber, which helps in the establishment of healthy bacteria and the production of SCFAs, which are very important in ensuring a healthy gut [70]. Consuming a balanced mixture of both animal and vegetable proteins is suggested to support a diverse and healthy gut microbiome. The addition of vegetable proteins and dietary fiber can alleviate the adverse consequences of excess protein fermentation and support gut health [66].

Animal models have suggested that protein quality can modulate the composition of the gut microbiota. For instance, a preclinical trial conducted by Zhang et al. [71] showed that cheese whey proteins enhanced fecal *Lactobacilli* and *Bifidobacteria* counts as opposed to casein. Mung bean protein also increased the abundance of the *Ruminococcacea* family in a model of mice fed a high-fat diet. Based on this, the bile acid metabolism of *Ruminococcacea* family members was thought to be healthier in high-fat-diet mice [72].

### 3.3. Fats

**Figure 6:**
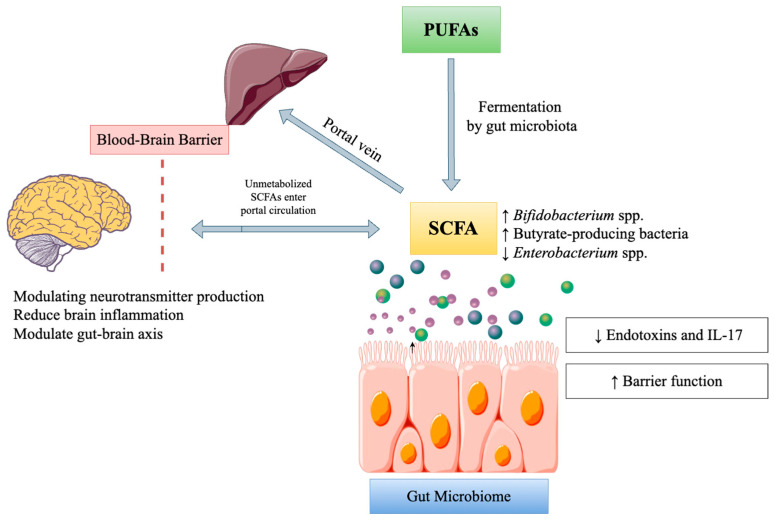
The role of PUFAs in relation to the gut microbiome and brain function. ↑, increase; ↓, decrease.

The roles of dietary lipids extend even to the microscopic organisms thriving within the human stomach. There have been differences observed with regard to the health of the gut and the type of fats consumed by hosts in different organisms [73]. Positive effects on the gut microbiome can be expected with the use of unsaturated fatty acids. Unsaturated fats are, on the other hand, friendly to the intestinal microflora. For example, a link has been found between omega-3 fatty acids, increases in anti-inflammatory bacteria, and decreases in the level of pathogenic bacteria [74]. Wolters et al. [75] also found a possible association between the availability of MUFAs (palmitoleic, oleic, and eicosenoic acids) and genera of the family *Enterobacteriaceae*—*Prevotella*, *Turicibacter*, and *Parabacteroides*. Conversely, research has established that diets abundant in MUFAs, especially those in peanuts, sesame, pumpkin seeds, and rapeseed oil, as well as extra virgin olive oil, result in an improvement in the microbial composition of both healthy and unhealthy mice, including those susceptible to metabolic syndrome. MCFAs present in human breast milk, infant formulas, and virgin coconut oil have the potential to facilitate the supplementation of *Bifidobacterium* and *Lactobacillus* and improve cognitive and metabolic functions [76]. Polyunsaturated fatty acids (PUFAs) are called “essential fatty acids” since the human body cannot generate them and must obtain them through the diet. The main sources of PUFAs include fatty fish, nuts, seeds, and sunflower oil. Omega-3 PUFAs can be advantageous by increasing the number of *Lachnospiraceae* taxa that produce butyrate and re-establishing the optimal bacterial ratio [57]. As shown in Figure 6, certain bacterial species become more abundant as a result of PUFA metabolism. Many metabolites, including SCFAs such as butyrate, are consequently generated, leading to a decrease in endotoxin and interleukin (IL)-17 production, which may benefit human health by reducing inflammation. PUFA metabolism produces unmetabolized SCFAs that enter the systemic circulation and exert immunoregulatory effects, especially with the brain, influencing gut–brain functions.

Diets high in saturated fats affect the gut microbiota in a negative way. These fats have the potential to decrease the number of good bacteria like *Bifidobacterium* and *Lactobacillus* while promoting the growth of hazardous ones. This imbalance, or dysbiosis, can lead to an increase in gut permeability, called “leaky gut”, promoting systemic inflammation and contributing to metabolic disorders [77].

### 3.4. Micronutrients

Several micronutrients, including zinc and omega-3 fatty acids, may exert a significant impact on the functions of the brain and on development without affecting the gut microbiota [78]. Since single nutrients are never eaten in isolation in real life, nutritional research has shifted away from concentrating on them. Instead, it has been demonstrated that the most advantageous diet for both physical and mental health consists of a wide variety of various nutritional foods [79]. Dietary nutrients that support normal brain function include vitamins, minerals, and amino acids [78,80]. Many of these nutrients help with cell signaling, myelination, neurotransmitter production, and metabolic processes, among others, by acting as cofactors for enzymes [81]. Omega-3 fatty acids, folate, s-adenosyl methionine, inositol, and vitamins B3, B6, and C are among the nutrients with antidepressant effects that have been the subject of extensive research. Supplementation with these nutrients may be advantageous, but only as an addition to a diet that promotes gut health [78]. Because of the fact that eicosanoids produced by AA have inflammatory qualities and eicosapentaenoic acid has anti-inflammatory properties, a high ratio of omega-6 to omega-3 fatty acids may cause a pro-inflammatory condition. In addition, evidence suggests that a lack of omega-3 fatty acids may be responsible for some psychiatric disorders, such as depression [82]. For example, B vitamins are necessary for the production of neurotransmitters and for the preservation of the myelin sheath, while zinc and magnesium are crucial for the release of neurotransmitters and for synaptic plasticity. These micronutrient deficiencies can also result in gut dysbiosis, an overgrowth or imbalance of gut bacteria that usually presents with inflammation and the disrupted production of neurotransmitters, thereby worsening depressive symptoms [81]. Calcium, magnesium, potassium, and iron can affect the microbiome. These minerals have been proven to increase levels of healthy bacteria such as Bifidobacterium and Lactobacillus, which improve gut health [83]. Too much iron can lead to germ overgrowth, whereas too little iron can reduce the function and the variety of microbes present [84]. Zinc deficiency can result in dysbiosis and increased susceptibility to infections. Zinc supports the integrity of the gut barrier and promotes a balanced microbial environment [83].

To sum up, evidence suggests that a diet rich in fiber, polyphenols, and micronutrients positively alters gut microbial composition, reduces metabolic endotoxemia and neuroinflammation, and is associated with better cognitive health. There are numerous small-scale observational and interventional studies that have shown the advantages of fiber for brain function and health [85]. Eating patterns also contribute to the control and release of serotonin produced in the ENS, with the most important factor being the intake of complex carbohydrates with tryptophan. Some components, including polyunsaturated fatty acids, B vitamins, zinc, and folate, which are dietary micronutrients, may also promote the healthy growth and development of the brain and its functions, while a lack of such nutrients in the diet causes the opposite effect by promoting mental disorders and aggravating brain diseases.

### 3.5. Phytochemicals and Bioactive Compounds

The utilization of phytochemicals and bioactive agents such as polyphenols, flavonoids, and other plant-based materials has gained interest concerning their impacts on the gut microbiota and the brain, especially on depression. These substances are present in large quantities in fruits, vegetables, tea, and other herbs and represent antioxidants, anti-inflammatory agents, and prebiotics, thereby affecting the gut–brain axis considerably. For instance, it is known that polyphenols (and other compounds found in berries, red wine, and dark chocolate) affect gut microbiome composition through the stimulation of populations of probiotic bacteria such as *Lactobacillus* and *Bifidobacterium* and the suppression of pathogenic strains [86]. The enhancement of microbial populations correlated with health promotes systemic immunity so that inflammation is lessened, assists in the repair and healing of the gut lining, and increases the absorption of SCFA-like butyrate, which has neuroprotective functions and is associated with better mental state outcomes [87].

Flavonoids, another group of bioactive compounds found in foods like citrus fruits, onions, and green tea, are also necessary for supporting gut health and mitigating depressive symptoms. These compounds have been shown to improve the abundance and diversity of good gut bacteria, contributing to a healthier microbiome [88]. The fermentation of flavonoids by gut microorganisms produces numerous metabolites that have the ability to directly impact brain function by passing across the blood–brain barrier, modulate neurotransmitter levels, and exert anti-inflammatory actions. For example, the flavonoid quercetin has been observed to increase levels of serotonin and dopamine in the brain, which are crucial for mood regulation [89]. Additionally, flavonoids can inhibit the activation of microglia, the brain’s resident immune cells, thereby reducing neuroinflammation, which is often elevated in individuals with depression [90].

Furthermore, the interplay between bioactive compounds and the gut microbiome can influence the gut–brain axis through the modulation of tryptophan metabolism. Tryptophan, an essential amino acid, is a precursor to serotonin, a neurotransmitter that plays a vital role in mood regulation [45]. Phytochemicals can impact the gut microbiota’s ability to metabolize tryptophan, shifting its metabolic pathways toward the production of serotonin and other beneficial metabolites rather than kynurenine, which is associated with neurotoxicity and inflammation. This shift can help alleviate symptoms of depression by enhancing serotonin availability and reducing inflammatory markers in the brain [91].

In summary, phytochemicals and bioactive compounds exert multifaceted effects on the gut microbiota, promoting the growth of beneficial bacteria, enhancing the production of neuroprotective metabolites, and modulating inflammatory responses. These changes in the gut environment can positively influence the gut–brain axis, contributing to improved mental health and offering a promising avenue for the dietary management of depression. Continued research on the specific mechanisms and optimal dietary sources of these compounds is essential for developing effective nutritional strategies to support mental health through gut microbiota modulation.

### 3.6. The Influence of Dietary Patterns and Components on the Gut Microbiome in Modulating Depression

The composition and function of the gut microbiome can be greatly impacted by specific dietary patterns, leading to changes in mental health, including the severity and occurrence of depression [78]. The Western diet has been linked to detrimental effects because of the heavy consumption of processed foods, red meat, refined carbohydrates, and saturated fats, which has been associated with negative impacts on gut microbiota and mental health. Usually, this diet causes an increase in harmful bacteria and a decrease in microbial diversity, leading to gut dysbiosis. The resulting imbalance in the gut microbiome can enhance intestinal permeability, promote systemic inflammation, and adversely affect the gut–brain axis. These changes are often linked to increased levels of pro-inflammatory cytokines and neuroinflammation, which are associated with the development and exacerbation of depressive symptoms [92]. In contrast, the Mediterranean diet, rich in fruits, vegetables, whole grains, nuts, seeds, and olive oil, along with the moderate consumption of fish and poultry, has been shown to promote a healthy gut microbiome and support mental health. This diet increases the abundance of beneficial bacteria such as *Bifidobacterium* and *Lactobacillus* and enhances microbial diversity. The high content of fiber and polyphenols in the Mediterranean diet supports the production of SCFAs such as butyrate, which has anti-inflammatory properties and can improve the integrity of the gut barrier. These changes help to reduce systemic and neuroinflammation, contributing to a lower risk of depression [93]. The relationship between vegan and vegetarian diets and mental health outcomes, particularly depression, is complex and has yielded mixed findings in recent research. These diets are typically high in fiber, vitamins, minerals, and phytonutrients, which promote microbial diversity and the development of advantageous gut microorganisms. The high fiber content encourages the synthesis of SCFAs, which have anti-inflammatory and neuroprotective properties. Some studies suggest that individuals adhering to plant-based diets may experience lower incidences of depressive symptoms [94]. Conversely, other research findings indicate a potential association between plant-based diets and increased depressive symptoms [95]. With these conflicting results [96], the current body of research does not provide a definitive conclusion. Further studies are necessary to clarify these associations and to understand the underlying mechanisms involved. The ketogenic diet, characterized by high fat, moderate protein, and very low carbohydrate intake, has shown mixed effects on the gut microbiome and mental health. While some studies suggest that the ketogenic diet can reduce inflammation and support mental health by modulating neurotransmitter levels and reducing neuroinflammation, others indicate that it may reduce microbial diversity and negatively affect gut health due to low fiber intake. The impact of the ketogenic diet on depression remains an area of active research, with some evidence supporting its use in specific cases, such as treatment-resistant depression [97]. In summary, dietary patterns play a crucial role in shaping the gut microbiome and influencing mental health. Diets high in polyphenols, fiber, and healthy fats, like the Mediterranean, support a healthy gut microbiome and are associated with a reduced risk of depression. Conversely, diets high in processed foods and saturated fats, like the Western diet, can negatively impact gut health and exacerbate depressive symptoms.

A number of biological systems linked to depression may be directly impacted by the Western diet and other diets associated with metabolic endotoxemia. A nutritious diet full of fresh fruits and vegetables is strongly associated with subjective happiness and mental health, according to numerous studies [80,98]. One of the first interventional studies on this topic was a 12-week, parallel-group, single-blind randomized controlled trial (RCT) including thirty-three male and female participants with moderate to severe depression. The participants were assigned at random to either social assistance or dietary help treatments. In order to promote adherence to the suggested diet, the dietary intervention included tailored dietary advice and nutritional counseling, as well as goal setting, mindful eating, and motivational interviewing [99]. Compared to conventional therapy alone, the study’s results showed that dietary intervention significantly reduced depression symptoms. This implies that changing the gut microbiota with an adjuvant dietary intervention may be a helpful way to treat depression.

In the MooDFOOD RCT, a diet fortified with calcium, vitamin D, selenium, and omega-3 fatty acids did not lessen major depressive episodes in people who were overweight or obese and had subsyndromal depressive symptoms [100], despite a recent RCT’s confirmation of the benefits of a Mediterranean-style diet for depression [101]. But taking into consideration only those participants who adhered for a full year, a supplemental diet may be able to delay the onset of depression. The HELFIMED trial looked into how food interventions directly affected participants’ self-reported depression. Patients who self-reported being depressed were put on a Mediterranean diet that included fish oil for a 6-month intervention period. To enable maximum dietary adherence, they also received nutritional coaching and cooking instruction. The control group received social support in order to take into consideration any possible non-dietary antidepressant benefits of nutritional counseling. The study’s findings showed a substantial decline in depression, which was positively connected with greater adherence to the Mediterranean diet during both 3- and 6-month periods. The study also revealed an intriguing correlation between a lower ratio of omega-6 to omega-3 fatty acids and a reduction in depressive symptoms [100]. In conclusion, the above-mentioned advantages of a diet high in plants for depression likely include improved intestinal permeability, which lowers metabolic endotoxemia, and anti-inflammatory effects mediated by increased polyphenol and SCFA synthesis. Increasing the consumption of omega-3 fatty acids and trace minerals may also help to address deficiencies in certain important nutrients related to mood and overall brain health.

Numerous dietary components that are linked to either a higher or lower incidence of depression also change the gut flora (Table 1). It is possible that a dietary component’s impact on gut microbiota may mediate its effect on mood, either fully or partially.

## 4. Potential Mechanisms Linking Diet, the Gut Microbiome, and Depression

### 4.1. Inflammatory Pathways

Progressive and persistent forms of depression are aggravated by inflammatory pathways, and more studies have provided evidence of the association between chronic inflammation and mood disorders. The complex triad of diet, the gut microbiome, and inflammation provides a hopeful opportunity to ameliorate neuroinflammation and depression. An altered diet seeking to modify the gut bacteria can have an extensive effect on inflammation as a result of the immune pathways targeted by bacterial metabolic products. These diet-induced bacterial metabolites, including, but not limited to, SCFAs, polyphenol metabolites, and secondary bile acids, have an effect on the production of pro-inflammatory and anti-inflammatory cytokines, which affect the CNS and also depression [2].

Increased levels of pro-inflammatory cytokines like IL-6, tumor necrosis factor-α (TNF-α), and IL-1β, which are implicated in starting inflammatory processes, are influenced by the CNS through several mechanisms that affect organs and, in turn, lead to depression [112]. The development of such a response is complicated further by the release of pro-inflammatory cytokines, which may also act on the HPA axis and increase the levels of cortisol. Studies have shown that the elevation of the stress hormone cortisol is linked with some psychiatric conditions, particularly mood disorders, cognitive deficits, and changes in the volume of mood-related brain areas such as the hippocampus and prefrontal cortex. Additionally, inflammatory cytokines have a direct effect on the bioavailability of critical neurotransmitters that are best known for their modulation of mood, mainly serotonin, dopamine, and glutamate [113]. Depressive symptoms are correlated with low levels of serotonin and an increase in neurotoxic metabolites like quinolinic acid, which leads to the overactivation of NMDA receptors, causing toxicity and consequent depressive symptoms [114]. Motivation and reward pathways, which are generally dysfunctional in depression, are also affected by chronic peripheral inflammation, which decreases dopamine and alters glutamate signaling [115]. The permanent inflammation of the peripheral system can lead to the activation of microglia, the immune system cells present in the brain, which leads to a state known as neuroinflammation. Activated microglia secrete inflammatory cytokines and generate ROS that are harmful to tissues in the brain, killing healthy neurons and inhibiting their regeneration [116]. The hippocampus, a functionally very important brain area in mood control and cognition, is most affected due to reduced neurogenesis, which has been associated with depressive conditions. Neuroinflammation also has implications for neuroplasticity; hence, it reduces the capacity of the brain to adjust and cope with stress and increases the severity of depressive states [117]. Inflammatory cytokines can reduce the levels of brain-derived neurotrophic factor (BDNF), a protein that promotes neurogenesis and supports the survival of neurons. BDNF is crucial for maintaining neuroplasticity and resilience to stress. Inflammatory markers like IL-1β inhibit BDNF production, particularly in the hippocampus, impairing neurogenesis and reducing the brain’s capacity for stress adaptation. This reduced neuroplasticity makes the brain more susceptible to depressive states and less responsive to treatment. Inflammatory processes contribute to oxidative and nitrosative stress, which further impacts brain function and mood regulation [118]. Inflammation affects the gut–brain axis and increases gut permeability (leaky gut), allowing inflammatory endotoxins like LPS to enter the bloodstream. LPS triggers systemic inflammation, which can reach the brain and activate the HPA axis, as well as neuroinflammatory pathways, contributing to depressive symptoms. The gut–brain axis thus serves as an important route by which inflammation originating in the gut can influence brain function and mood [119].

A high-fiber diet is particularly effective in reshaping the microbiome to support anti-inflammatory functions by producing SCFAs. Butyrate plays a dual role: it acts directly on the gut epithelium to enhance tight junction integrity, reducing endotoxemia, and also interacts with immune cells to decrease the secretion of pro-inflammatory cytokines. Butyrate binds to G-protein-coupled receptors (GPCRs), specifically GPR41 and GPR43, which are expressed on colonic epithelial and immune cells, activating anti-inflammatory pathways through downstream signaling cascades such as the inhibition of the NF-κB pathway. This, in turn, suppresses microglial activation in the CNS, thereby modulating neuroinflammation associated with depressive symptoms [45]. Polyphenols, naturally present in a wide range of plant foods like berries, tea, and cocoa, undergo extensive metabolism by gut bacteria, yielding bioactive metabolites that cross the blood–brain barrier and exert neuroprotective effects. For example, gut-derived metabolites of polyphenols, such as urolithins (derived from ellagic acid) and phenyl-γ-valerolactones (from flavan-3-ols), have been shown to inhibit pathways that generate pro-inflammatory mediators like IL-1β and TNF-α through the suppression of the JAK/STAT and MAPK pathways in immune cells. These metabolites also support antioxidant defenses by upregulating nuclear factor erythroid 2–related factor 2 (Nrf2) signaling, which reduces the oxidative stress that often coexists with neuroinflammatory conditions [120]. Importantly, polyphenol metabolites modulate the gut–brain axis by preventing the activation of peripheral immune cells that contribute to neuroinflammation, thereby potentially reducing depressive symptoms [121]. Fang et al. [122] also highlighted the role of alpha-linolenic acid, eicosapentaenoic acid, and docosahexaenoic acid in regulating gut microbiota to counter inflammatory processes. Along with food, omega-3 PUFAs, also commonly known as “healthy oils”, are transformed into specialized pro-resolving mediators (SPMs), which include resolvins and protectins. These SPMs are features of distinct cells and target specific G proteins in immune cells. SPMs promote inflammation resolution and decrease the level of pro-inflammatory cytokine production [123].

Linking the availability of SCFAs, polyphenolic metabolites, and SPMs with the diet-modified microbiome provides an indication of precise mechanisms by which gut microbiome dietary modulation may alter inflammation in depression. Strategies aiming to modify the composition of the gut microbiome in an anti-inflammatory manner to enhance metabolite barriers and change the composition of the microbial community will have little net impact on the levels of pro-inflammatory cytokines such as IL-6 and TNF-α, which are known to aggravate depression. This approach to reducing inflammation provides a compelling foundation for dietary interventions aimed at treating or preventing depressive disorders, supporting the targeted modulation of gut-derived inflammatory pathways to improve mental health outcomes.

### 4.2. Neurotransmitter Synthesis

The gut microbiome is a major contributor to the production and modulation of various neuroactive substances, including serotonin, dopamine, and GABA, which have been proven important for mood and behavioral control [124]. The gut microbiome influences the generation of neurotransmitters using several mechanisms, including the production of the precursors of the neurotransmitters, the modulation of enzymes involved in neurotransmitter production, and the gut–brain axis. The microbiome can, therefore, modulate the peripheral and central nervous systems’ neurotransmitter levels, mood and stress fluctuations, and psychiatric disorders (like depression and anxiety).

#### 4.2.1. Serotonin Production

Approximately 90% of the serotonin in the human body is produced within the gastrointestinal tract, and certain gut bacteria are known to regulate its synthesis [33]. Various species, such as *Enterococcus* and *Streptococcus*, have been found to generate metabolites that promote the release of serotonin from enterochromaffin cells present in the gut lining [125]. This peripheral serotonin is not able to cross the blood–brain barrier, although it is known to assist with gut movement, immunity, and enteric nervous system function, among others. Tryptophan, a precursor of serotonin, is also influenced by certain microbial metabolites like SCFAs, which in turn affects the amount of serotonin produced. An optimal gut microbiota allows for more conversion of tryptophan to serotonin and less conversion to the neurotoxic products of the kynurenine pathway, which cause depression [126]. In addition, the stimulation of the microbial biosynthesis of SCFAs such as butyrate is effective in strengthening the gut lining, which in turn decreases peripheral inflammation and helps to maintain serotonin signaling within the central nervous system [125].

#### 4.2.2. Dopamine Synthesis

Dopamine is known to play a key role in the regulation of reward, motivation, and mood states, and determining how the gut microbiota may also play a role is an area of active research. Some types of bacteria, such as *Bacillus* and *Escherichia coli*, have been shown to either synthesize dopamine or influence its synthesis from precursors such as tyrosine and phenylalanine [125]. Dopamine synthesized in the gut holds significance in mediating enteric nervous system signaling mechanisms, which in turn affect the motility and immune responses of the gut. The “gut–brain axis” also allows microbial metabolites, such as SCFAs, to reach the brain and modulate the dopaminergic system. For example, SCFAs are known to affect the function of the brain’s reward center, where even a slight distortion leads to depressive symptoms, as the mesolimbic dopaminergic neurons are responsible for dopamine production. Also, microbial populations in the gut or their absence can alter the permeability of the blood–brain barrier, inhibiting or permitting the passage of dopamine precursors into the circulation and ultimately the brain, where they can increase the synthesis of dopamine, responsible for improving mood and increasing motivation [127].

#### 4.2.3. GABA Production

As a neurotransmitter, GABA competes against the stimulative effects of natural brain activity. It is critical in decreasing levels of anxiety and facilitating relaxation, as well as moderating mood. Some gut microorganisms, for example, *Lactobacillus* and *Bifidobacterium*, synthesize GABA [125]. A part of the GABA synthesized in the gut is likely to be released to the brain via the vagus nerve, which connects the brain and the gut. Research has demonstrated that changes in the gut microbiome can lead to changes in GABA receptors in the brain associated with mood and stress changes. Specifically, it has been shown that the supplementation of *Lactobacillus rhamnosus* leads to increased GABA receptors in the amygdala and prefrontal cortex, which influences anxiety behavior. Additionally, this GABA modulation achieved via bacteria in the gut may also offset the “elevated” levels of the neurotransmitter “glutamate” associated with depression and anxiety [128].

#### 4.2.4. Gut–Brain Communication and Neurotransmitter Modulation

The microbiome affects the levels of neurotransmitters that can be found in the brain mainly because of the gut–brain axis, which is a two-way communication channel that utilizes neural, hormonal, and immunological mechanisms to signal between the two organs. Such compounds as SCFAs, secondary bile acids, and derivatives of tryptophan have access to the ventral vagal nerve and then to the CNS, where they participate in the production and function of different neurotransmitters and their receptors. It has been observed that these interactions influence the level of BDNF, a neuroprotective factor that promotes adaptation in stress-provoking conditions and neuroplasticity. Changes in BDNF and neurotransmitter production are brought about by the gut microbiome, which, therefore, is capable of influencing the neuroplasticity of an individual, leading to changes in mood or behavior [91].

### 4.3. HPA Axis Modulation and the Gut Microbiome’s Role in Stress Response

The HPA axis is at the core of how the body responds to stress, especially with the production of cortisol, which has a wide range of effects, including mood regulation, immune function, and protecting the body from stress and its effects. The gut microbiome is an important factor in determining the activity of the HPA axis in relation to stress, both in terms of its onset and maintenance. When stress is experienced by the body, the hypothalamus exerts a direct influence over the pituitary gland’s activity, which induces the adrenal glands to secrete cortisol, the primary function of which is to help the body cope with stress. Cortisol enables the individual to cope with stress for a short duration; however, at times, it can cause an overdrive of the HPA axis, which is frequently triggered by prolonged stress or inflammation. HPA axis dysregulation then occurs, which is linked with mood disorders, especially anxiety and depression [88].

The HPA axis is also affected by the gut microbiota, in most cases by more than one mechanism. Some beneficial gut microflora, for example, species of *Lactobacillus* and *Bifidobacterium*, have been shown to exert protective effects against HPA activation caused by stress, at least in part by acting on the vagus nerve, which serves as a major part of the gut–brain axis. These microbes secrete SCFAs and other molecules that affect the immune and hormonal systems to prevent excessive inflammation of the HPA axis, which would lead to its excessive stimulation [129]. Furthermore, gut dysbiosis is associated with changes in gut function, such as increased gut permeability, which causes the translocation of endotoxins such as LPS into the circulation, evoking an immune response. The presence of increased levels of LPS then causes the activation of the HPA axis, which leads to the pathological production and accumulation of cortisol, which has been implicated in depression, inflammatory disorders of the gut, and issues linked to neurodegeneration [59].

Furthermore, LPS and peptidoglycan, which serve as microbe-associated molecular patterns (MAMPs) produced by microorganisms, along with the subsequently produced pro-inflammatory mediators, activate the HPA axis. The primary bacterial cell wall component, known as peptidoglycan, has an adverse effect on behaviors and brain development because it penetrates the blood–brain barrier and triggers the innate immune system’s pattern recognition receptors. Similarly, LPS derived from gut microbes and peptidoglycan induces the activity of NOD1, a protein involved in the activation of the immune system [130].

The HPA axis and stress mechanisms involve interactions with different macro- and micronutrients. The consumption of diets rich in refined sugars and saturated fats has been found to correlate with elevated cortisol levels and altered HPA axis functioning [131]. Cortisol elevation and stress response failure caused by the deficiency of certain micronutrients like magnesium and vitamin B6 have been documented [132]. One of the beneficial effects of omega-3 fatty acids is the regulation of the HPA axis, which is manifested as reduced cortisol levels and an enhanced ability to cope with stress. Madison et al. [133] performed a meta-analysis and reported that cortisol levels were significantly decreased with omega-3 supplementation, with more effective stress responses overall. Such effects are thought to be connected with inflammation regulation via omega-3s, which shows an HPA axis-related effect.

### 4.4. Oxidative Stress and Neuroprotection

As defined, oxidative stress is an excess accumulation of ROS, which becomes detrimental since the body cannot detoxify them adequately and is a major contributor to the pathogenesis of mood disorders, especially neurovegetative disorders, e.g., depression and anxiety. Elevated oxidative stress factors can evoke neuronal injury and provoke inflammation and the disruption of neurotransmission, all of which are associated with mental health disturbances. The gut microbiome helps in enhancing oxidative stress and neuroprotection by producing metabolites, altering the immune system, and controlling the mechanisms of antioxidants [134].

Specific bacteria situated within the gut produce SCFAs such as acetate, propionate, and butyrate, which perform functions that involve being anti-inflammatory and being rich in antioxidants that inhibit oxidative stress. For example, butyrate aids in maintaining the structure of the blood–brain barrier, preventing the entry of ROS and peripheral inflammatory markers into the central nervous system [135]. Furthermore, complex fatty acids extend the activity of antioxidant enzymes such as superoxide dismutase (SOD), which decomposes ROS alongside glutathione peroxidase, thus shielding neurons against the harmful effects of oxidative stress. Moreover, some gut bacteria synthesize BDNF, a neuroactive compound that is important in coping with stress and promotes neuroplasticity [136].

On the other hand, dysbiosis of the gut refers to an alteration in the normal gut microflora; this is characterized by excess oxidative stress, both within the gut and outside it. Gut dysbiosis is therefore characterized by increased intestinal permeability, which paves the way for the entry of pro-inflammatory agents such as LPS into the circulation, resulting in inflammatory responses and inducing oxidative stress [45]. This excessive oxidative stress can also compromise neuroprotection and disturb the balance of the nervous system by causing neurodegeneration, which is one of the factors that contribute to the development of various mood disorders. This implies that maintaining a healthy balance of gut microbes helps the body manage oxidative stress with ease and provides neuroprotection, which highlights the relationship between gut health, oxidative stress, and mental health.

### 4.5. Depression-Related Changes in the Abundance of Gut Microbes

In recent years, there has been an increased interest in assessing the correlation between gut microbiome diversity and mental health, more specifically, depression. The existing research findings indicate that both the diversity and composition of gut microbiota contribute to the modulation of mood and mental well-being. Healthy gut microbiome diversity entails the composition and quantity of different microbial organisms present within the GI tract. It has been documented that higher diversity generally leads to healthier outcomes, such as a functional immune response and a regulated metabolic state. On the other hand, the decreased diversity of the microbiome has been shown to be related to several health challenges, such as gastrointestinal diseases, metabolic syndrome, and psychological disorders [64].

Research on germ-free animals, or animal models without sophisticated gut microbiota, has shed greater clarity on the relationship between gut microbiota and host neurobehavior. A reduction in *Parabacteroides* and *Bacteroides* was linked to an increase in immune-regulating genes and pro-inflammatory cytokines in a rat prenatal stress model. Anxiety developed as a result of these alterations, causing systemic inflammatory reactions [137]. Early life stress has been shown to increase the number of *Oscillibacter*, *Parasutterella*, *Treponema*, *Ruminiclostridium*, and *Helicobacter* while decreasing that of *Bacteroides*, *Rikenellaceae*, *E. ruminantium*, *Lactobacillus*, and *Parabacteroides*. Elevated corticosterone, adrenocorticotrophic hormone, and glucocorticoid receptor levels in the hippocampal region are linked to these alterations. These changes in the composition and number of gut microbes inhibit miR-124a and promote the production of miR-132. Furthermore, the increased expression of N-methyl-D-aspartate receptor (NR2A and NR2B) and glucocorticoid receptors was more frequent in rats treated with α-amino-3-hydroxy-5-methyl-4-isoxazolepropionic acid receptors (GluR1 and GluR2) [138]. Numerous studies have connected anxiety and depressive behaviors to the pathogenic influence of gut dysbiosis [124,139]. Patients with depression often express symptoms of GI disturbances, including bloating, nausea, vomiting, abdominal discomfort, and constipation [140]. Depression pathology is connected with the disproportionate abundance of *Enterobacteriaceae* and *Alistipes* species (increased), as well as *Faecalibacterium* species (reduced) [102]. The presence of pathogenic microbiota families, including *Ruminococcaceae*, *Shewanellaceae*, *Halomonadaceae*, and *Verrucomicrobiae*, was positively connected with patients’ anxiety-like behavior, whereas the *Lachnospiraceae* and *Bacteroidaceae* families were associated with both anxiety and IBD [141]. The possible connection between gut dysbiosis and depressive-like behavior is clearly suggested by these clinical data (Table 2).

## 5. Interventional Studies: Modifying the Diet to Influence Mental Health Through the Gut Microbiota

### 5.1. Dietary Interventions

To a greater degree than would have been the case some years ago, research now shows that dietary and eating patterns have an effect on mental well-being—especially depressive states. Adherence to dietary patterns such as the Mediterranean diet, characterized by the consumption of vegetables, fresh fruits, cereals and grains, nuts, and olive oil, is associated with a lower prevalence of depression, probably due to the high antioxidant properties of the above foods. Many of these foods seem to help lessen age-related oxidative stress, inflammation, and intestinal dysbiosis and promote favorable neurochemical processes. Following an average follow-up of 20.4 years among 49,261 Swedish women, those who strictly adhered to a Mediterranean diet pattern had a lower incidence of depression [151]. The Mediterranean diet is associated with improved gut microbial diversity, decreased GI inflammation, and intestinal barrier integrity. Two important biochemical connections between the Mediterranean diet and gut dysfunction are propionate and butyrate, two SCFAs produced by the microbiota [152]. On the flip side, the Western diet, which emphasizes processed food with high levels of added sugar, fats, oils, and other refined products, has been associated with an increased prevalence of depression. This dietary pattern may exacerbate the risks of inflammation, gut dysbiosis, and neurotoxicity, which have been found to be depressive. Also, it has been shown in several longitudinal studies and meta-analyses that adherence to a Western diet is associated with more mood disorders than Mediterranean and other health-promoting diets.

### 5.2. FMT (Fecal Microbiota Transplantation)

FMT is a viable intervention technique for chronic diseases linked with dysbiosis, since it is a quick way to reshape the patient’s gut microbiota through the administration of fecal flora from healthy donors. It is well known from preclinical research that FMT can reduce depressive-like behavior. Mice with alcohol-induced depression-like behavior were cured when the microbiota from healthy donors was transplanted. Additionally, rats with stress-induced depression exhibited improved phenotypes due to neuroinflammation suppression, gut microbiota imbalance correction, and intestinal barrier restoration [153]. Bacteriophages may mediate the effectiveness of FMT [154]. Apart from the global microbiota transplantation performed during the FMT method, *Lactobacillus plantarum* mono-colonization shields *Drosophila* from depressive-like states [155]. The gut–brain axis may rely on the vagus nerve as a key signaling pathway to control the protective effects of FMT on depression. Regardless of the resolution of GI symptoms, FMT treatment progressively reduced depression symptoms in patients with diarrhea-predominant IBD [112]. An RCT further confirmed these beneficial treatment outcomes in patients with concurrent IBD, anxiety, and depression [69]. In a different RCT, the effectiveness of frozen oral FMT capsules was evaluated as a further treatment for MDD patients, and it was discovered that four weeks following transplantation, depressive symptoms considerably improved [156]. FMT therapy for depression also reduces GI symptoms and rebalances the gut environment, much like it does for autism. Despite negative effects and complications of FMT therapy being documented in some articles, this treatment approach is becoming more and more popular in clinical and scientific settings. This is especially the case for an alternative pill made from human feces that produces similar effects in a less invasive and more standardized manner [157].

### 5.3. Probiotics, Prebiotics, and Synbiotics

Recent clinical trials have shown that probiotics, prebiotics, and synbiotics may positively influence depression by modulating the gut–brain axis. When taken in sufficient quantities, living bacteria, known as probiotics, are beneficial to the health of the host. Probiotics containing beneficial bacteria, like *Lactobacillus* and *Bifidobacterium*, have shown reductions in depressive symptoms, possibly through anti-inflammatory and neurotransmitter-modulating effects [158]. As evidenced in Table 3, numerous studies have shown the effectiveness of different probiotic species (e.g., *L. helveticus*, *L. rhamnosus*, *B. longum*, and *B. breve* CCFM1025) in the treatment of clinical depression. It is crucial to remember that some research has produced unfavorable findings [159,160]. These contradictory results could be the result of heterogeneity in the cohort characteristics and/or the therapies employed, although this is unknown. Additionally, the synergistic effects of several species can boost the antidepressant effects of probiotics. According to Liu et al. [161], this implies that utilizing multi-species probiotics may be more advantageous than using single-species probiotics.

Prebiotics are substrates that the gut bacteria preferentially use to support host health. They may encourage the growth of certain advantageous microorganisms. Prebiotics, such as fructooligosaccharides (FOSs) and galactooligosaccharides (GOSs), promote beneficial bacterial growth and may improve mood by enhancing gut health [161]. Probiotic (*L. helveticus* and *B. longum*) and prebiotic (GOS) supplementation’s effects on depression remission in patients with MDD were examined in a previous RCT. After eight weeks of treatment, it was found that probiotics, but not prebiotics, reduced the symptoms of depression [162]. In an RCT, administering patients with depression with 4G-β-D-galactosylsucrose for 24 weeks consistently increased their sense of self-efficacy without affecting their depressive symptoms [163]. By encouraging the growth of probiotics, prebiotics indirectly improve host health rather than having a direct impact on the body.

Synbiotics—combinations of probiotics and prebiotics—have shown promise as well, with some studies indicating enhanced effects on mood compared to those of probiotics alone. A synbiotic supplement (containing *L. acidophilus*, *L. casei*, and *B. bifidum* plus inulin) improved depressive symptoms in overweight and obese adults [164]. Probiotics and prebiotics have symbiotic connections. Thus, it is conceivable that synbiotics will lead to the next advances in the treatment of depression. It is thought that administering probiotics and prebiotics at the same time increases the activity of good gut bacteria. However, the careful selection of appropriate prebiotics and probiotic strains is necessary for the synthesis of successful synbiotics. Moreover, outcomes vary based on formulation, dosage, and individual gut microbiota composition, highlighting a need for further standardized research. Table 3 highlights the evidence from recent studies on prebiotic, probiotic, and synbiotic interventions.

**Table 3:** Recent prebiotic, probiotic, and synbiotic intervention studies.

Intervention	Dose	Study Design	Outcome	References
Probiotic studies—preclinical studies				
*Lactobacillus reuteri* NK33 and *Bifidobacterium adolescentis* NK98	1 × 10^9^ CFU for 5 days	Immobilization stress-induced depression in C57BL/6 mice	↓ Proteobacteria population and gut LPS production ↓ IL-6 and corticosterone levels ↓ Anxiety and depression phenotypes	[165]
*Lactobacillus kefiranofaciens* ZW3	1 × 10^7^ CFU, 1 × 10^8^ CFU, and 1 × 10^9^ CFU for 2 weeks	Kunming male mice: CMS model of depression	↑ Stress-induced serum corticosterone levels ↑ Brain 5-HT levels ↑ Depression-like behavior	[166]
*Lactobacillus helveticus* MCC1848	1 × 10^11^ CFU ml^−1^ for 24 days	Male C57BL/6J (B6) mice: Subchronic and mild social defeat stress (sCSDS) model of depression	Restored normal sucrose consumption Restored nucleus accumbens dopamine and serotonin receptor gene expression	[167]
*Bifidobacterium breve* CCFM1025	1 × 10^9^ CFU ml^−1^ for 5 weeks	Male adult C57BL/6 mice: CMS model of depression	↑ Metagenomic tryptophan biosynthesis and profile of SCFA producing bacteria Normalized the stress-induced expression of brain Nr3c1 glucocorticoid receptor ↑ BDNF and precursor levels	[168]
Clinical studies				
Probiotic (*Lactobacillus helveticus* and *Bifidobacterium longum*) and prebiotic (galactooligosaccharide)	1 × 10^10^ CFU per 5 g sachet for 8 weeks	Patients with MDD: RCT for psychological outcomes	↓ Depression score ↓ Kynurenine/ tryptophan ratio	[162]
*Bifidobacterium longum* 1714	1 × 10^9^ CFU ml^−1^ for 4 weeks	Randomized, double- blind, parallel-group design: Social stress-induced by “Cyberball” game	↑ Theta band power and beta-3 band power in cortex in resting stage Changes in the neural processing of social stress	[169]
Prebiotic studies—preclinical studies				
Fructooligosaccharide from *Morinda officinalis*	50 mg kg^−1^ for 3 weeks	Male SD rats: 7-week CMS model of depression following 4 weeks of treatment	Recovery of sucrose intake ↑ Mobility and exploratory behavior ↓ Plasma corticosterone Repaired damage in the intestinal epithelium	[170]
Synbiotic studies—clinical studies				
Synbiotic formulation 15 g of prebiotics and 5 g of probiotic containing *L. acidophilus* T16 and *B. bifidum* BIA-6,7,8	2.7 × 10^7^ CFU g^−1^ each for 12 weeks	75 Hemodialysis patients: Serum BDNF was measured	↓ Clinical anxiety and depression scores ↑ Serum BDNF levels	[171]

Abbreviations: ↑, increase; ↓, decrease.

## 6. Limitations and Challenges

One of the major limitations in utilizing dietary interventions to manage depression is the significant variability in individual responses. This variability can arise from numerous factors, including genetic differences, baseline microbiome composition, lifestyle factors, and the presence of other health conditions. For instance, while some individuals may experience significant mood improvements and gut microbiome benefits from dietary changes, others may see little to no effect or even negative outcomes, highlighting the need for personalized dietary recommendations. Additionally, the long-term sustainability of dietary interventions poses another challenge. While short-term dietary changes can be beneficial, maintaining these changes in the long term can be difficult due to factors such as dietary preferences, socio-economic barriers, cultural influences, and the complexity of adhering to specific dietary regimens. This sustainability issue is compounded by the often restrictive nature of some therapeutic diets, which can lead to decreased adherence and potential nutrient deficiencies if not carefully managed. Thus, for dietary interventions to be effective in the long term, they must be feasible, culturally acceptable, and enjoyable, ensuring that individuals can maintain the recommended dietary patterns without feeling overly restricted or deprived.

## 7. Gaps in the Knowledge and Future Research Directions

### 7.1. The Need for Longitudinal and Large-Scale Studies

Despite the growing evidence linking diet, gut microbiome composition, and mental health, there is a pressing need for more longitudinal and large-scale studies to fully understand these complex relationships. Current research often relies on cross-sectional data or short-term interventions, which can provide only a snapshot of the interactions between diet, the microbiota, and mental health. Longitudinal studies are crucial for observing how changes in dietary patterns influence the gut microbiome and mental health outcomes over time, offering insights into causality rather than mere association. Additionally, there is a significant gap in the research regarding diverse populations. Many existing studies are conducted on homogeneous groups, limiting the generalizability of the findings across different ethnicities, ages, genders, and socio-economic backgrounds. Understanding the variations in dietary impacts across diverse populations can help to identify specific dietary recommendations tailored to different demographic groups, thereby enhancing the effectiveness and applicability of dietary interventions for mental health, particularly depression.

### 7.2. Mechanistic Studies

To advance our understanding of how diet influences mental health through the gut–brain axis, it is essential to delve deeper into the precise molecular mechanisms involved. Mechanistic studies are needed to elucidate the specific pathways by which dietary components affect the gut microbiota and how these microbial changes translate into alterations in brain function and mood regulation. For example, while we know that certain diets can increase the production of SCFAs and modulate tryptophan metabolism, the exact molecular interactions and regulatory pathways remain unclear. Understanding these mechanisms at a detailed level, including the role of specific microbial species, metabolites, and host factors, can help in the development of targeted therapies and interventions. Additionally, mechanistic studies can explore the interplay between diet-induced microbial changes and other physiological systems, such as the immune and endocrine systems, which are also implicated in the pathophysiology of depression. This comprehensive understanding is critical for identifying new therapeutic targets and optimizing dietary strategies for mental health improvement.

### 7.3. Personalized Nutrition

The idea of personalized nutrition represents a future perspective considering the feasibility of dietary management strategies based on an individual’s microbiome and genetic constitution. Since the results of dietary interventions vary across individuals, personalized nutrition solutions can offer some dietary recommendations based on the gut microbiota composition, metabolic potential, and genetic factors of the individual. This is easy in theory because it involves mapping out how different foods and food patterns affect different individuals and what impact those individual effects have, if any, on mental health. In the present study, emphasis is placed on future projections concerning the emergence of effective and producible technologies providing a basis for predictive anthropometric strategies utilizing microbiome data. This can be seen as a large potential future step in the practical application of nutrition therapy, as it accepts that there are differences among individuals; therefore, it is not promoting a blanket approach. The last but most fundamental tool to be adopted for this will be advanced technologies such as the exploration of metagenomics and metabolomics, as well as the use of machine learning in understanding the interplay between food, microbiome composition, and mental health.

## 8. Conclusions

The interdependence between the diet, the gut microbiome, and depression emphasizes the importance of nutrition in mental health. Various dietary fibers, proteins, fats, and other nutrients are consumed by gut microbes and converted into different metabolites: SCFAs, indoles, and precursors of neurotransmitters, the effects of which can be seen on the gut–brain axis and, in turn, mood and cognitive abilities. Such metabolites produced by microbes have the ability to affect inflammation, the production of neurotransmitters, and the integrity of the gut barrier, all of which are important in understanding the mechanisms that cause depression. Especially interventions that include changes in fiber and omega-3s in the diet or the general use of a healthy balanced diet have been effective in positively impacting mental health through colonizing the gut with healthy bacteria and curtailing inflammation in the body. However, the differential effects on individuals and the compliance with these interventions for prolonged periods remain constraints. Future studies must be longitudinal or mechanistic and include functional strategies such as personalized nutrition in order to enable researchers to grasp the benefits of the gut microbiome for the treatment of mental disorders. The evidence calls for the incorporation of dietary modalities as a part of integrative mental healthcare beyond the use of medications, with the hope that nutrition and gut health will become the mainstay in curbing or treating depression in the future.

## Figures and Tables

**Table 1:** Examples of dietary patterns linked to depression that have an immediate impact on the host while also interacting with the gut microbiome.

Dietary Component	Study Model	Effect	Reference
High-carbohydrate diet	18 SPF male C57BL/6J mice, 6–8 weeks old	Increased depression Increased levels of circulation and central nervous system inflammatory responses Abnormal expression of central 5-HT and its receptors Significantly decreased the diversity of intestinal flora	[102]
Dietary capsaicin	Male C57BL/6J mice, 4 weeks old	Reduced depression Relative abundances of *Ruminococcus* and *Prevotella* Increased levels of the monoamine neurotransmitter 5-HT Reduced levels of the inflammatory cytokine TNF-α	[103]
Herbal extracts	Male C57Bl6/J mice, 8 weeks old	Reduced depression Changes in the gut microbiome Increased SCFA production	[104]
Dietary fiber	Patients with hypertension from the Hospital of Soochow University and Jinchang Community, China	Reduced depression Protective effect on depression and anxiety	[105]
Dietary fiber	Male CD-1 mice, 3 months old	Reduced depression Maintained the integrity of the gut barrier Regulated the structure of the gut microbiota Production of related metabolites	[106]
Dietary fiber	Female C57BL/6J mice, 2 months old	Reduced depression Enhanced SCFA generation Restructured the gut microbiome	[107]
Apple polyphenol	Male C57BL/6 mice, 3–4 weeks old	Ameliorated depression Increased the richness and diversity of the gut microbiota Increased relative abundance of *Firmicutes* and *Bacteroidota* Decreased relative abundance of *Verrucomicrobiota* at the phylum level	[108]
Phenols	C57BL/6J mice, 5 weeks old	Reduced depression Regulated the structure of the gut microbiota Regulated microbial metabolism products such as 5-HTP	[109]
Oolong tea polyphenols	Male C57BL/6J mice, 6–8 weeks old	Reduced depression Reshaped intestinal microbiota dysbiosis Regulated cognition-related metabolites Strengthened mucosal integrity and intestinal barrier dysfunction by increasing tight junction protein expression	[110]
Orange flavonoids	Participants from Seoul and Gyeonggi-do, age 20–30 years	Reduced depression Changed the relative abundance of the gut microbiome, especially the butyrate-producing *Lachnospiraceae* family.	[111]

**Table 2:** Alterations in gut microbial abundance under depressive conditions.

Depression Model	Genus						Sample Site
	*Lactobacillus*	*Bacteroides*	*Clostridium*	*Bifidobacterium*	*Allobaculum*	*Turicibacter*	
CUMS	↑ [142]	↑ [143]	↑ [143]	↑ [144]	↑ [144]	-	Cecum
	↓ [145]	↓ [145]	↓ [146]	↓ [147]	-	↓ [146]	Fecal pellets
CRS	↓ [148]	↓ [148]	↓ [149]	↓ [149]	↓ [149]	↓ [148]	Fecal pellets
MS	↓ [138]	↓ [138]	↓ [138]	-	-	↓ [138]	Fecal pellets
CSDS	↓ [150]	-	-	↓ [150]	↓ [150]	↓ [150]	Fecal pellets

Abbreviations: CUMS, chronic unpredictable mild stress; CRS, chronic restraint stress; MS, maternal separation; CSDS, chronic social defeat stress; -, not investigated, ↑, increase; ↓, decrease.

## Abbreviations

The following abbreviations are used in this manuscript:

5-HT	5-hydroxytryptamine
BCFA	Branched-chain fatty acid
BDNF	Brain-derived neurotrophic factor
CNS	Central nervous system
CRS	Chronic restraint stress
CSDS	Chronic social defeat stress
CUMS	Chronic unpredictable mild stress
ENS	Enteric nervous system
FMT	Fecal microbiota transplantation
FOS	Fructooligosaccharides
GABA	Gamma-aminobutyric acid
GALT	Gut-associated lymphoid tissue
GI	Gastrointestinal
GLP	Glucagon-like peptide
GOS	Galactooligosaccharide
HPA	Hypothalamic–pituitary–adrenal
IBD	Inflammatory bowel disease
IDO	Indoleamine-2,3-dioxygenase
IL	Interleukin
LPS	Lipopolysaccharide
MDD	Major depressive disorder
MUFA	Monounsaturated fatty acid
MS	Maternal separation
PUFA	Polyunsaturated fatty acid
RCT	Randomized controlled trial
ROS	Reactive oxygen species
SCFA	Short-chain fatty acid
SPM	Specialized pro-resolving mediators
TNF-α	Tumor necrosis factor-alpha
